# Einführung zur Qualität von Medizinjournalismus und erste Einschätzungen zur COVID-19-Berichterstattung

**DOI:** 10.1007/s00103-020-03249-x

**Published:** 2020-11-30

**Authors:** Dominik Daube, Georg Ruhrmann

**Affiliations:** Institut für Kommunikationswissenschaft, Friedrich-Schiller-Universität Jena, Ernst-Abbe-Platz 8, 07743 Jena, Deutschland

**Keywords:** Journalismus, Digitalisierung, Expertenkommunikation, Framing, Evidenz, Journalism, Digitization, Expert communication, Framing, Evidence

## Abstract

Die Medienlandschaft unterliegt einem stetigen Wandel, innovative Medientechnologien beeinflussen in immer stärkerem Maße unsere Lebenswelt. Da digitale Medien zunehmend in allen Altersklassen konsumiert werden und Laien sich im Internet auch häufig zu gesundheitlichen und medizinischen Themen informieren, stellt sich die Frage nach der Qualität dieser (journalistischen) Inhalte im Web. Die Rolle von Journalistinnen und Journalisten wandelt sich und für die Wissenschaft gibt es neue Möglichkeiten und Herausforderungen bei der digitalen Verbreitung von Forschungsergebnissen. Welchen qualitativen Ansprüchen müssen diese Inhalte genügen und welche Potenziale und Chancen, aber eben auch Risiken bringen online verbreitete und rezipierte Informationen mit sich?

Im vorliegenden Beitrag werden Themen der Medizin- und Gesundheitskommunikation und Prozesse der journalistischen Berichterstattung beschrieben. Anschließend diskutieren wir das in der Medizin bereits etablierte Kriterium der „Evidenz“ als möglichen Ansatz zur Beurteilung gesundheitsrelevanter und medizinischer Informationen. Abschließend wird die COVID-19-Pandemie im Kontext des wachsenden Medienpopulismus diskutiert.

## Abstract (en)

The media landscape is subject to constant change; innovative media technologies increasingly influence the world we live in. While digital media are increasingly being consumed by all age groups and laypersons frequently obtain information on health and medical topics on the Internet, the question of the quality of (journalistic) content on the Web arises. The roles of journalists are changing and scientists are experiencing new opportunities and challenges in the digital distribution of research results. Which qualitative requirements have to be met by these contents and which potentials and opportunities, but also risks, do online disseminated and received information entail?

This article describes the topics of medical and health communication and the processes of journalistic reporting. Subsequently, we discuss the criterion of “evidence,” which is already established in medicine, as a possible approach to the evaluation of health-relevant and medical information. Finally, we discuss the corona pandemic in the context of growing media populism.

## Einleitung

Die zunehmende Etablierung innovativer Medientechnologien vor allem in den letzten Jahrzehnten hat zu erweiterten (kommunikativen) Potenzialen von (Massen‑)Medien geführt [1]. Aus historischer Sicht gibt es schon seit Langem immer wieder wichtige Innovationen im Medienbereich. Sie reichen – um nur einige zu nennen – vom informierenden Charakter der Zeitung und ihrer Nachrichtengebung im 18. Jahrhundert [2] über die Telegrafie bis zur kommerzialisierten TV-Unterhaltung [3]. Doch die aktuellen Entwicklungen der individualisierten und partizipativen Kultur im Social Web lassen sich als ein neuer Meilenstein begreifen [4].

Der Konsum von digitalen Medieninhalten steigt rasant an, über 50 Mio. Menschen nutzen in Deutschland täglich das Internet, 41 % der Bürgerinnen und Bürger greifen auf mediale Internetangebote wie Video-on-Demand-Plattformen, klassische Mediatheken oder digitale Ausgaben von Zeitungen zu [5]. Sehr häufig suchen und rezipieren die Nutzerinnen und Nutzer dabei medizinische Informationen [6, 7]. Somit ist das Internet mit den online jederzeit und überall verfügbaren Informationsquellen zu einem der wichtigsten Kanäle bei der Informationsbeschaffung geworden [8]. Glaubwürdigkeit, Korrektheit und Präzision von medial präsentierten Inhalten können dabei stark variieren, Informationen können frei und potenziell von jeder Person mit einem Internetzugang geteilt und konsumiert werden.

Dies birgt neben dem großartigen Potenzial des freien Zugangs zu medizinischem Wissen und Nachrichten auch das Risiko, insbesondere bei hohem Medienkonsum [9] fehlerbehaftete Inhalte (z. B. Fake News, Furcht und Verschwörungstheorien) zu internalisieren und weiter zu verbreiten [10, 11]. Ein Mindestmaß an Medienkompetenz, besser noch ein Grundverständnis wissenschaftlicher Arbeitsweisen und Gütekriterien, sind Voraussetzung für eine effiziente Informationsrezeption.

Dieser Beitrag hat das Ziel, überblicksartig die Probleme aufzuzeigen, die sich durch die Digitalisierung ergeben, mit Schwerpunkt auf die Berichterstattung zu gesundheitlichen und medizinischen Themen. Gefragt wird, welche Qualitätskriterien von der journalistischen Berichterstattung erfüllt werden (sollen) und wie sie zu bewerten sind. Ist die Evidenz der mitgeteilten medizinischen Aussagen dabei als ein potenzieller Lösungsansatz zu sehen? Das aktuelle Beispiel der COVID-19-Berichterstattung im Kontext von Medienpopulismus verdeutlicht, wie drängend und auch schwierig die Frage nach der Qualität geworden ist.

## Digitalisierung als Herausforderung für den Gesundheitsjournalismus

Die akademische bzw. hier relevante kommunikationswissenschaftliche Erforschung neuer medialer medizinischer Informationsangebote ist wesentlich Gegenstand der Gesundheitskommunikation. Dieses inter- und auch transdisziplinäre Forschungs- und Lehrgebiet [12] beginnt sich – ausgehend von den USA und dem Vereinigten Königreich – auch im deutschsprachigen Raum als zunehmend eigenständige Disziplin an den Hochschulen zu etablieren [13].

Auch abseits von Ausnahmesituationen wie der aktuellen Pandemie hat die Gesundheitskommunikation sich medizinischen und gesundheitsbezogenen Themen angenommen, wie etwa Debatten über Impfungen/Impfpflicht, Organspende, Lebensmittelsicherheit oder andere spezifische Krankheiten. Die Wahrnehmung dieser Themen und der Verlauf der Debatten werden häufig durch eine intensivierte journalistische Berichterstattung und die parallel laufende Kommunikation im Social Web maßgeblich beeinflusst [14].

### Informationssuche in klassischen Medien und online

Die Rollen der Akteurinnen und Akteure in der sich digitalisierenden Medizinberichterstattung verändern sich, indem sie sich neuen bzw. veränderten ökonomischen, politischen, rechtlichen und auch soziokulturellen Rahmenbedingungen des Gesundheitssystems anpassen und sich vor allem auch mit den wissenschaftlich-technischen Fortschritten der modernen molekularen Medizin auseinandersetzen [15].

Die Digitalisierung ist zunächst durch den barrierearmen Zugang zu zahlreichen Onlineangeboten gekennzeichnet. Immer mehr Zeitungen bieten ihre Inhalte zusätzlich online an, dieses Angebot ist zu einem Großteil kostenfrei zugänglich: So hat sich die Zahl der Onlineangebote seit 1999 um mehr als das Vierfache auf 698 Angebote in Deutschland gesteigert, nahezu die Hälfte aller Zeitungsleserinnen und -leser nutzt sowohl das Print- als auch das Onlineangebot parallel [16]. Außerdem bieten die neuen, crossmedialen Strategien der klassischen Medien ein hohes Maß an Interaktionspotenzial über Social-Media-Kanäle sowie die Möglichkeit des direkten Feedbacks über Kommentarfunktionen in den Onlineangeboten von Zeitungen und Fernsehsendern.

Für die Rezipierenden ist es schwierig, die sehr zahlreichen und diversen Informationsangebote qualitativ zu bewerten und zu selektieren. Es ist bisher noch unklar, wie genau diese Bewertungsprozesse ablaufen. Studien weisen darauf hin, dass bislang häufig Inhalte rezipiert werden, die eigeninitiativ gar nicht gesucht werden würden [17].

Bei medizinischen Fragen sind Ärztinnen und Ärzte auch im digitalen Zeitalter noch die präferierte Informationsquelle. Aber auch die klassischen Massenmedien sind weiterhin relevant [18]. Inwieweit die Informationen hier allerdings intendiert gesucht werden, ist häufig schwer nachvollziehbar. Denn zahlreiche Formate liefern zwar relevante Inhalte, sind aber nicht explizit darauf ausgelegt. Auch ist im Vorhinein nicht erkennbar, dass bestimmte Inhalte präsentiert werden. Offengelegt und bewusst intendiert wäre die Informationsrezeption beispielsweise bei einer Talkshow zu einem bestimmten Gesundheitsthema (z. B. Organspende) – hier ist die Thematik für jedermann ersichtlich. Weniger transparent sind die Themen in Unterhaltungsformaten, beispielsweise Arztserien. Sie können ein Thema in einen Handlungsstrang aufnehmen, ohne dass das Publikum gezielt danach gesucht hat. Ähnlich verhält es sich bei Printmedien, auch hier können in verschiedenen Formaten Themen mit Gesundheitsbezug journalistisch verarbeitet werden, ohne dass es für die Leserschaft sofort ersichtlich wird. Die folgenden Abschnitte werden sich dieser Thematik widmen.

### Selektion von Medieninhalten und Meinungsbildung

**Figure 1:**
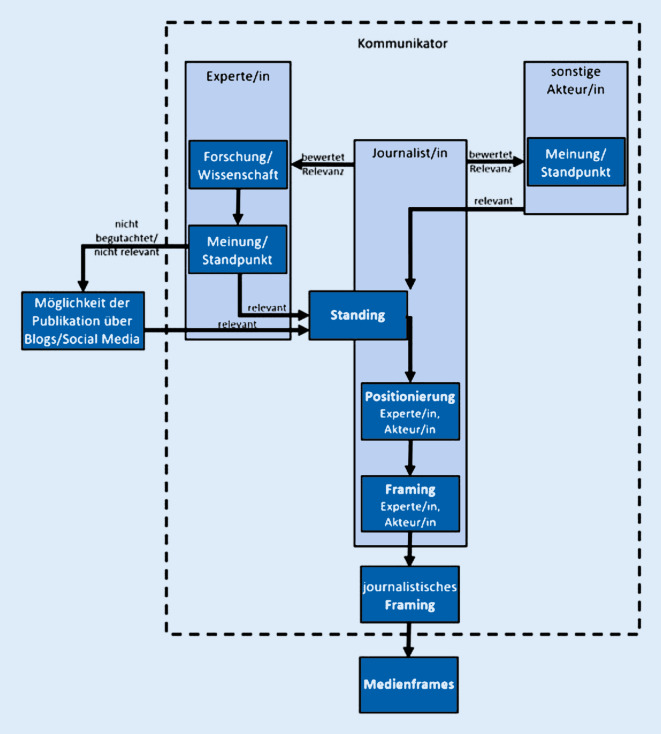

Wenn Menschen nun Informationen zu bestimmten Gesundheitsthemen und zur Medizin suchen, stellt sich die Frage, welche Angebote ihnen (in den Massenmedien) zur Verfügung gestellt werden. Journalistinnen und Journalisten übernehmen eine wesentliche Selektionsfunktion und entscheiden somit, welche Inhalte in den Medien auf die Agenda gesetzt und wie sie berichtet werden. Zunächst muss dabei der *Anlass der Berichterstattung *betrachtet werden, da er durch die Wissenschaft initiiert oder auch durch andere gesellschaftliche Bereiche bestimmt werden kann. Akteurinnen und Akteuren lassen sich dabei unterschiedliche Dimensionen des medialen Erfolgs [19] zuweisen, etwa zunächst das Standing, was bedeutet, dass ein Inhalt die journalistische Selektion erfolgreich durchlaufen hat und öffentlich platziert werden kann („zu Wort kommen“; Abb. 1). Entscheidet eine Journalistin oder ein Journalist, dass die Expertenmeinung relevant ist und sie journalistisch bearbeitet werden soll, so ist das Standing erfolgreich (Abb. 1).

Im nächsten Schritt (Positionierung) geht es um die Bewertung eines wissenschaftlichen Themas (z. B. konkret der Medizin in den Medien) durch die Akteurinnen und Akteure (z. B. Medizinexpertin oder Medizinexperte). Haben sich diese erfolgreich platzieren können (Standing erfolgreich), so ist nun entscheidend, welche Position sie oder er gegenüber der Thematik einnimmt (Abb. 1). Das behandelte Thema kann negativ, positiv, ambivalent oder neutral beurteilt werden.

Gesundheitsrelevante Inhalte können medial sowohl intendiert als auch nichtintendiert als „Beiwerk“ verbreitet werden. Der kommunikationswissenschaftliche Ansatz des Framings setzt sich in diesem Zusammenhang mit der Rahmung von Informationen und Inhalten auseinander. Eine verbreitete Definition dieses Ansatzes geht auf Entman zurück: „To frame is to select some aspects of a perceived reality and make them more salient in a communicating text, in such a way as to promote a particular problem definition, causal interpretation, moral evaluation, and/or treatment recommendation for the item described“ [20]. Entmans Ausführung folgend können (medizinische) Themen in den Medien unterschiedlich dargestellt werden, indem sie in bekannten und routinemäßig verwendeten Deutungsmustern rekonstruiert werden (Medienframes). Die Art und Weise der Darstellung wirkt sich auf die Informationsverarbeitung des Rezipierenden aus, es werden bestimmte Schemata (z. B. Meinungen) ausgebildet (Rezipientenframes).

**Figure 2:**
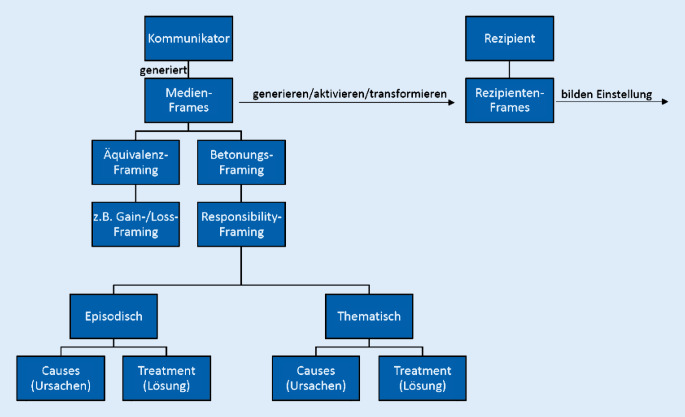

Das Medienframing kann u. a. in *Äquivalenz- und Betonungsframing* unterteilt werden [21]. Äquivalenzframing meint die unterschiedliche Rahmung eines identischen Inhalts (Glas halb voll vs. Glas halb leer), beide Rahmungen sind gleich informativ. In der Gesundheitskommunikation wird in diesem Kontext vor allem das Gewinn- und Verlustframing untersucht: Es fokussiert auf die Vor- oder Nachteile einer bestimmten Handlung oder eines Objekts ([22, 23]; Abb. 2), beispielsweise eines Medikaments oder einer Therapie.

Beim *Betonungsframing* wählen die Verfasserinnen und Verfasser die Information inhaltlich vorab aus und nennen gezielt bestimmte Fakten, andere Daten werden bewusst ausgespart [20, 21]. Dieses Framing ermöglicht also eine strategische Kommunikation, die den öffentlichen Diskurs gezielt beeinflussen kann [24]. So wird beispielsweise während der aktuellen COVID-19-Pandemie die Verantwortlichkeit bestimmter (privater wie öffentlicher) Akteurinnen und Akteure für den Ausbruch der Pandemie oder deren Eindämmung medial unterschiedlich aufbereitet. Im Journalismus werden dabei auch nichtmedizinische bzw. allgemein unwissenschaftliche Deutungen und Interpretationen zugelassen [19, 25]. Außerdem lassen sich unterschiedliche Akteurinnen und Akteure (und deren Handlungen) sowohl als Verantwortliche für das Entstehen eines Missstandes einerseits oder für die Lösung eines bestimmten Problems andererseits ausmachen. Diese Verantwortungszuschreibung lässt sich mit „Responsibility-Frames“ analysieren. Diese lassen sich aufgrund ihrer gezielten Nennung (oder Aussparung) von verantwortlichen Akteurinnen und Akteuren als eine Form des *Betonungsframings* kategorisieren (Abb. 2).

Der Ansatz des Responsibility-Framings hat seinen Ursprung in der Unterscheidung von *thematischen* und *episodischen* Frames von Iyengar [26]. Thematische Frames betonen abstrakt gesellschaftliche Entwicklungen mittels aufbereiteter Werte (z. B. Darstellung über Diagramme) oder indem Inhalte in einen gesamtgesellschaftlichen Kontext (z. B. historisch) eingeordnet werden und begünstigen damit eine Verantwortungszuschreibung auf gesellschaftlicher Ebene, z. B. durch die Nennung statistisch aufbereiteter Kennzahlen wie der Inzidenz oder Prävalenz einer Erkrankung in der Gesellschaft. Episodische Frames sind gekennzeichnet durch individuelle Erlebnisse und Erfahrungsberichte einzelner Betroffener und führen zu einer tendenziellen Verantwortungszuschreibung auf individueller Ebene, wenn beispielsweise ein Erkrankter als Einzelfall in einem Beitrag porträtiert wird, nimmt der Lesende eher das individuelle Schicksal wahr und bedenkt ein mögliches strukturelles (gesamtgesellschaftliches) Problem dahinter weniger oder gar nicht. Ferner unterscheidet Iyengar zwischen „Ursache“ und „Lösung“, die letztlich von Semetko und Valkenburg im Jahr 2000 als „Responsibility Framing“ identifiziert wurden: „This frame presents an issue or problem in such a way as to attribute responsibility for its cause or solution to either the government or to an individual or group“ [27].

An dieser Stelle sei nochmals darauf hingewiesen, dass Framing sowohl intendiert stattfinden kann – Journalistinnen und Journalisten einen Beitrag also gezielt aufbereiten – oder sie ihn nicht gezielt rahmen. Letzteres tun sie, wenn sie gesundheitsrelevante und medizinische Inhalte kommunizieren und beispielweise mit Fallbeispielen aufbereiten. Kessler et al. untersuchten die intendierte Rahmung der Fernsehberichterstattung im Medizinjournalismus und identifizierten 3 Frames: neue wissenschaftliche Verfahren in der molekularen Medizin, Grundlagenberichterstattung und kritische Reflexion aktueller Forschungsdefizite in der molekularen Medizin [28].

Eine neue Studie im Kontext von Responsibility-Framing untersuchte die gesamte Berichterstattung – inklusive nichtintendierter Rahmungen. Analysiert wurden dort die medial verbreiteten Risikofaktoren und Behandlungsverantwortungen bei Demenzerkrankungen [29]. Die Studie zeigte, dass die Verantwortungszuschreibung für die Erkrankung selbst auf individueller Ebene stattfindet, während die Behandlung eher auf gesellschaftlicher Ebene zu verorten ist. Dies kann einen starken Einfluss auf die Meinungsbildung der Rezipierenden haben, wobei die entsprechenden Informationen jedoch nicht sämtlich gezielt verbreitet wurden, sondern sich auch abseits der Medizinkommunikation aus der Gesamtheit der Berichterstattung ergeben.

### Qualitätsbewertung

Wie aber können durch die medizinische Wissenschaft kommunizierte Inhalte hinsichtlich ihrer Qualität bewertet werden? Und in welchem Verhältnis stehen die Akteurinnen und Akteure des Journalismus und der medizinischen Forschung zueinander, vor allem dann, wenn es um die öffentlichkeitswirksame Kommunikation von Medizinerinnen und Medizinern geht, also gerade nicht um die Kommunikation innerhalb einer (klinischen) Fachöffentlichkeit (wissenschaftlicher Diskurs)? Wie bereits erwähnt, entscheiden in den klassischen Massenmedien Journalistinnen und Journalisten, welche Inhalte und Statements veröffentlicht werden und somit die öffentliche Meinung beeinflussen können.

Gerade mit der Digitalisierung öffnen sich für die Medizin neue Möglichkeiten, Inhalte mit der breiten Öffentlichkeit zu teilen: So kann und wird über Blogs oder andere soziale Medien (z. B. Twitter) außerhalb der Fachöffentlichkeit kommuniziert [30, 31]. Die Abhängigkeit der Wissenschaft von Journalistinnen und Journalisten nimmt ab, da die Instanz der journalistischen (Qualitäts‑)Begutachtung und Filterung, das klassische Gatekeeping (Torwächterfunktion), entfällt (siehe unten). Gerade die damit (mehr oder weniger ausgeprägt) stattfindende Qualitätssicherung wäre aber für eine genaue und richtige, vollständige und ausgewogene, transparente und vor allem evidenzbezogene Berichterstattung (siehe unten) essenziell, um einen hohen Standard zu sichern. Der Deutsche Presserat hat hierzu einen ausführlichen Kodex erstellt, nach dessen Grundsätzen die Journalistinnen und Journalisten sowie die publizierende Presse handeln sollen [32]. Für den Gesundheits- und Medizinjournalismus gibt es im Pressekodex sogar eine eigenständige Ziffer (Ziffer 14), welche vor fälschlicherweise Furcht oder Hoffnung schürenden Sensationsdarstellungen warnt und eine explizite Kennzeichnung von ungesicherten Forschungsergebnissen fordert [32]. Geht eine schriftliche Beschwerde gegen einen publizierten Artikel – online wie Print – beim Presserat ein, wird diese dort geprüft. Stellt er Verstöße gegen diesen Kodex fest, so können diese mit mehreren Maßnahmen geahndet werden: von einer öffentlichen Rüge, welche im betroffenen Medium abgedruckt werden muss, über die nichtöffentliche Rüge bis zur Missbilligung oder dem Hinweis [33]. Aus Platzgründen wird an dieser Stelle das Verfahren nicht detailliert behandelt.

Diese Richtlinien gelten gleichermaßen für Print- wie Onlinepublikationen, finden jedoch bei frei publizierten Inhalten ohne die zwischengeschaltete journalistische Instanz wenig bis keine Berücksichtigung mehr. An dieser Stelle soll trotzdem eine weitere Kontrolleinrichtung erwähnt werden, welche sich – vergleichbar mit dem Deutschen Presserat – der Kontrolle der Einhaltung ethischer Grundsätze und der Ahndung von öffentlichem kommunikativen Fehlverhalten verschrieben hat: der Deutsche Rat für Public Relations (DRPR e. V.; [34]). Er reagiert ebenfalls auf Beschwerdeeinreichungen, wird nach Bedarf aber auch eigeninitiativ tätig und kann nach der Begutachtung des jeweiligen Falls eine Mahnung oder Rüge veröffentlichen [34]. Seine Wirksamkeit wird später im Beitrag diskutiert.

### Ein möglicher Lösungsansatz – Evidenz?

Eine Grundfrage in der Gesundheitskommunikation lautet: Inwieweit werden gesundheitsrelevante bzw. medizinische Informationen vor der Weitergabe wissenschaftlich geprüft? Welche Kriterien der Belegbarkeit müssen medizinische Inhalte erfüllen, um – nicht zuletzt auch ethisch – vertretbar öffentlich kommuniziert werden zu dürfen? Wie sollten die Inhalte kommuniziert werden? Schließlich kann gerade im gesundheitlichen Kontext die Weitergabe ungesicherter oder falscher Informationen aus der Medizin zu einer akuten Gesundheitsgefährdung führen. Bleibt man nun bei den medizinjournalistischen Beiträgen, so ist ein denkbarer Lösungsansatz die evidenzorientierte Berichterstattung. Hier sei erwähnt, dass das Evidenzkriterium [35] in diesem Beitrag als zentrales Kriterium aus den zahlreichen Qualitätskriterien des Journalismus ausgewählt wurde. Andere gängige Kriterien (z. B. Vielfalt, Transparenz) können an dieser Stelle nicht vertieft behandelt werden (siehe dazu Beitrag von Anhäuser et al. in diesem Themenheft).

Während die Evidenzbasierung in der Medizin bereits eine lange Tradition hat und dort mittlerweile ein fester Bestandteil ist, bewegt sich nun auch die Gesundheitskommunikation in diese Richtung, konkret in die Richtung der *evidenzbasierten Gesundheitsinformation* [36]. Diese befasst sich primär mit der Aufbereitung und den Inhalten von Gesundheitsinformationen für Patientinnen und Patienten, lässt sich aber durchaus auch auf die inhaltliche und formale Aufbereitung journalistischer Texte anwenden. Hierzu zählt inhaltlich beispielsweise die Darstellung von Metastudien und Reviews, welche die höchste Qualität im Sinne der Evidenzpyramide aufweisen [37]. Formal wären beispielsweise die Darstellung von Zahlen oder die grafische Aufbereitung von Informationen zu nennen. In einer qualitativ hochwertigen journalistischen Berichterstattung ist es darüber hinaus relevant, auf die aktuellen Forschungserkenntnisse und dabei explizit auch auf die wissenschaftlich noch ungesicherten Befunde und Forschungslücken einzugehen. Mit anderen Worten: Die in der Wissenschaft normale konfligierende Evidenz, gegebenenfalls aber auch unterschiedliche wissenschaftliche Standpunkte sind zu repräsentieren [38, 39]. So wird auch eine möglichst komplexe und wirklichkeitsnahe mediale Darstellung ermöglicht [40]. Bisher werden Beiträge im Gesundheitsjournalismus nur selten mit wissenschaftlicher Evidenz belegt. Außerdem werden Inhalte, die mit wissenschaftlichen Belegen untermauert werden, noch immer häufig und traditionell als wissenschaftlich gesichert deklariert, auch wenn aus wissenschaftlicher Perspektive noch Unsicherheiten bestehen [41].

Bei der direkten Kommunikation von Experten über soziale Medien gestaltet sich die Einordnung schwieriger. Denn hier prüft nicht zwingend eine übergeordnete Instanz im Sinne eines Gatekeepers die vermittelten Inhalte vorab. Es sind die individuellen medizinischen und medienbezogenen Kompetenzen der Rezipierenden, beziehungsweise die der Anbieter der sozialen Netzwerke selbst gefragt. Falschmeldungen oder gezielte Fehlinformationen könnten durch die Anbieter sanktioniert und gegebenenfalls offengelegt werden (z. B. durch weiterführende Hinweise bei fragwürdigen Beiträgen), wobei die Wirksamkeit dieser sogenannten Label (gut sichtbare Kennzeichnungen) nach aktuellem Stand noch unklar ist [42]. An dieser Stelle würde also den sozialen Medien die Position einer Kontrollinstanz zugewiesen. Welche Mittel zur Kontrolle probat sind, bedarf aber noch weiterer Forschung.

## Aktuelles Beispiel: Berichterstattung zur COVID-19-Pandemie

Neben der Kontrolle der Korrektheit und der gerade angesprochenen Evidenz als Qualitätskriterium kommunizierter und berichteter Inhalte werden die sich stark wandelnden Verbreitungsstrategien (Kanäle, Zielgruppen) zunehmend relevant. Zwar birgt die Digitalisierung das Potenzial eines demokratisierten freien und offenen Zugangs zu und Austauschs von wissenschaftlichen Erkenntnissen. Doch das Beispiel COVID-19 verdeutlicht auch die Herausforderungen, die sich durch zunehmende Falsch- und Desinformationen bzw. einen – allerdings noch detaillierter zu untersuchenden – Medienpopulismus ergeben. Diese können die geschilderten Entwicklungen von Qualitätsjournalismus bzw. Evidenzorientierung möglicherweise konterkarieren.

Da die Pandemie im Gange ist und wir jetzt mit hoher Dynamik (exponentielles Wachstum der Infektionsfälle) am Beginn der „zweiten Welle“ stehen, kann keine abschließende Beurteilung erfolgen. Nicht wenige der (vor-)berichteten Studien sind zwar häufig evident, haben aber dennoch – wie die Autorinnen und Autoren selbst bemerken – einen vorläufigen Charakter und werden häufig ein paar Monate später ergänzt.

Trotz der naturgemäßen Unvorhersehbarkeit ist auch in diesen besonderen Situationen die rasche Kommunikation von Informationen essenziell, um die Öffentlichkeit zu schützen, aufzuklären und zu alarmieren [43]. Während der Anfangsphase der COVID-19-Pandemie gab es eine Informationsflut über zahlreiche Kanäle. Das Fernsehen lieferte regelmäßig Sondersendungen, die Onlineangebote der Printmedien richteten Liveticker ein, welche minütlich aktuelle Entwicklungen in der Pandemie listeten [44].

Zunehmend lassen sich dabei auf den großen Plattformen, wie z. B. Facebook oder Twitter, (wissenschaftliche) *Desinformationen *und *Falschinformationen* finden, etwa zum Verlauf der Pandemie, zur Wirkung von Hygieneregeln und Masken bis hin zu Impfstoffen. Dabei können die *meist absichtslos erstellten Falsch*informationen über SARS-CoV‑2 und den damit verbundenen Ausbruch von COVID-19 die Bürgerinnen und Bürger eher dazu veranlassen, weitere Informationsquellen zu suchen und behördlich verordnete Regeln zu befolgen, während die *absichtsvoll und gezielt eingesetzte Des*informationen – quasi der Gegenbegriff zu Falschinformationen – eher dazu führen können, dass weitere Informationen gemieden bzw. nicht gesucht werden und auch offizielle bzw. staatliche Anordnungen ignoriert bzw. nicht befolgt werden [45]. Das ist bedeutsam, gerade weil nicht wenige Inhalte (aus sozialen Netzwerken) bei entsprechenden Faktenchecks als „falsch“ eingestuft werden [46]. Auch zeigt sich, dass polarisierende Inhalte, die über sogenannte Social Bots (menschliches Verhalten imitierende Programme) in sozialen Netzwerken verbreitet werden, häufig auch mit rechtsextremen und verschwörungstheoretischen Plattformen und Seiten verbunden sind (vgl. [44, 47]).

Die Entwicklungen werden auch in der COSMO-Studie (COVID-19 Snapshot Monitoring) aufgegriffen, welche während des Ausbruchsgeschehens unter anderem das Informationsverhalten und die Akzeptanz für die Maßnahmen untersuchte [48]. Die häufigsten Bezugsquellen für Informationen waren während der ganzen Zeit die öffentlich-rechtlichen Medien, auch die Onlineausgaben von Zeitungen wurden relativ häufig konsultiert, während soziale Medien vergleichsweise selten verwendet wurden. Während zu Beginn noch häufig gezielt nach Informationen gesucht wurde, nahm der Informationsdrang ab Ende April tendenziell ab. Die Informationsflut könnte nach aktuellen Erkenntnissen zu Beginn zwar zu einer Sensibilisierung für die Thematik, recht bald jedoch zu einer Art Informationsüberdrüssigkeit und -müdigkeit geführt haben. Maßnahmen wurden in der Bevölkerung zunehmend kritisch betrachtet und die Forderung nach Lockerungen nahm zu.

Relevant sind daher die bekannten Merkmale von Medienpopulismus [49] und es ist zu prüfen, wie und inwieweit die Coronaberichterstattung davon betroffen ist:

- *Ingroup-Favorisierung*: Hier geht es um die Stereotypisierung von Eigen- und Fremdgruppe: Etwa im März 2020, als knappe Schutzausrüstung und Personalmangel der Gesundheitsämter sichtbar wurden und von Politik und Medien ständig relativ undifferenziert das deutsche Gesundheitssystem mit anderen Systemen in Südeuropa oder in den USA verglichen wurde [50–52]. Oder auch generationenspezifische Erfahrungen im Umgang mit neuen und neuartigen Risiken der Coronapandemie [53].
- *Elitenorientierung*: Unschwer zu erkennen ist aktuell eine Experten- und damit verbunden auch eine Eliteorientierung der Berichterstattung – weitgehend kommen hier männliche wissenschaftliche Experten und Spitzenpolitiker sowie Unternehmer und weniger die „systemrelevanten“ Ärztinnen und Ärzte, Krankenpflegerinnen und -pfleger oder Verkäuferinnen und Verkäufer zu Wort. Auch ist für soziale Medien zu zeigen, wie die Nutzerinnen und Nutzer die sogenannte Elite aus Angehörigen der Medizin, Wirtschaft und Politik wahrnehmen und beschreiben und wie sich das auf ihr Verständnis von Qualität und Evidenz auswirkt.
- In Bezug auf den vermeintlich „gesunden Menschenverstand“ sind in der Pandemieberichterstattung alle möglichen Formen jeweils eigener und für selbstverständlich gehaltener Alltagstheorien, Alltagswissen („Corona ist doch nur Grippe“), Alltagsnormen, vor allem Fatalismus [54], Gerüchte [55] und Verschwörungstheorien [56] über SARS-CoV‑2, seinen Ursprung und seine Bekämpfung zu beobachten. Dabei werden jeweils die eigenen (Gruppen‑)Normen als der gesunde Menschenverstand ausgegeben, insbesondere auch in Beiträgen im Netz [57]. Den anderen, der Fremdgruppe – in diesem Falle China – wird im Netz offen rassistischer Hass entgegengebracht [58].
- *Moralisierungen*. Gut bekannt sind für (Fernseh‑)Nachrichten seit Langem die moralisierenden, emotionalisierenden und personalisierenden Darstellungen [59]. Unterstützt werden solche Wertungen durch eine formal-strukturelle Rahmung der gezeigten Story (episodisches Framing). Vor allem gezeigt bzw. stark visualisiert werden Akteurinnen und Akteure und einfache Ursache- und Wirkungszusammenhänge, die auf individueller Ebene verortet werden [60, 61].

Ein aktuelles Beispiel in dieser moralisierenden Debatte ist die medienwirksame Auseinandersetzung zwischen dem Virologen Christian Drosten und der BILD-Zeitung [62]. Hier übte ein Medium Kritik an einer Expertenmeinung und setzte eine Diskussion über die Verlässlichkeit von wissenschaftlichen Aussagen in Gang. Während der wissenschaftliche Diskurs auf einen kritisch-rationalen Umgang setzt und durch ständige gegenseitige Kritik und Hinterfragen den Fortschritt und eine hohe Qualität – auch bei einer aktuell hohen Dynamik von Veröffentlichungen [63] – weitestgehend sicherstellt, kann dies bei Laien dazu führen, dass sie durch öffentlich ausgetragene wissenschaftliche Debatten verunsichert werden [64, 65]. Daher sollte journalistisch kritisch reflektiert mit der öffentlichkeitswirksamen Darstellung solcher Sachverhalte umgegangen werden, um einer Überinterpretation oder völligen Fehlinterpretation seitens der Rezipierenden vorzubeugen (wissenschaftliche Erkenntnisse werden durch Laien als gesichert und endgültig verstanden).

Ein weiteres Beispiel aus der Pandemie zeigt aktuelle Entwicklungen und Maßnahmen im Umgang mit virologischen bzw. medizinischen Inhalten in den sozialen Medien: die Rüge, die der Agentur „Storymachine“ durch den Deutschen Rat für Public Relations (DRPR) erteilt wurde [66]. Hier wurde eine mangelnde Neutralität bei der Informationsweitergabe kritisiert und durch den DRPR bekannt gemacht. Somit nahm der Rat – vergleichbar mit dem Deutschen Presserat – eine freiwillige (Selbst‑)Kontrollfunktion wahr und konnte die Öffentlichkeit informieren. An dieser Stelle muss jedoch auch erwähnt werden, dass dem Rat, wie bereits beschrieben, zusätzlich zu den öffentlichkeitswirksamen Bekanntmachungen über seine eigenen Kanäle keine härteren Sanktionierungsmöglichkeiten zur Verfügung stehen. Voraussetzung für die Wirksamkeit einer Rüge (oder ähnlicher Maßnahmen) ist also, dass diese Informationen bzw. Bekanntmachungen durch die Massenmedien (freiwillig) aufgegriffen und kommuniziert werden.

## Diskussion und Fazit

Inwiefern der soziale Wandel und die Digitalisierung den Journalismus beeinflussen, bedarf weiterer Untersuchungen. Eine interessante Forschungsgrundlage bieten die aktuellen Geschehnisse rund um COVID-19. Die Reaktionsrate der Akteurinnen und Akteure steigt durch die Vernetzung zunehmend, auch die Reichweite erhöht sich über die Kanäle der sozialen Medien deutlich: So können Expertinnen und Experten – wie bereits beschrieben – unmittelbar auf journalistische Beiträge eingehen, ohne vorab eine journalistische Selektion zu durchlaufen. Gleichzeitig birgt diese Art der Kommunikation auch das Risiko von Missverständnissen und Fehlinterpretationen. Expertinnen und Experten verlagern Diskussionen aus der wissenschaftlichen Gemeinschaft in öffentlich rezipierte Debatten, Laien verfügen häufig jedoch nicht über ausreichende Wissenschaftskompetenz, um diese Informationen ungefiltert korrekt einordnen zu können. Als „Lektionen“ [67] ergeben sich: Besonders wichtig sind professionelle und transparente (mediale und politische) Kommunikation, politische Führung mit eindeutigen und einheitlichen Aussagen und eine globale und verantwortungsbewusste Solidarität.

Eine symbiotische Beziehung gehen an dieser Stelle der Journalismus und die Expertinnen- und Expertenkommunikation über soziale Medien ein: Journalistische Beiträge betten Originalaussagen der wissenschaftlichen (wie auch politischen) Akteurinnen und Akteure von Plattformen wie Twitter oder Facebook ein, was zu einer neuartigen Kommunikation über diese Kanäle führt. Hier werden Originalaussagen journalistisch aufbereitet und die öffentlich geführte Debatte wird bestenfalls in einen für Laien verständlichen Kontext eingeordnet. So ergänzen sich die Vorteile der unmittelbaren öffentlichen Kommunikation über neue und wandelnde wissenschaftliche Erkenntnisse seitens der Expertinnen und Experten mit den Vorteilen journalistisch aufbereiteter Informationen.

Betrachtet man die Informationszugänglichkeit im Internet genauer, so lassen sich einige Bestrebungen ausmachen, medizinische Informationen qualitätsorientiert, also laienverständlich *und* evidenzbasiert aufzubereiten und so die Rezipierenden besser zu informieren und aufzuklären [68]. Eine solche unabhängige Informationsquelle, die bereits seit 2006 online ist und nach den Grundsätzen evidenzbasierter Gesundheitsinformation arbeitet, stellt das Onlinegesundheitsportal „gesundheitsinformation.de“ dar [69]. Auch erscheint es bedeutsam, angesichts der nicht abgeschlossenen COVID-19-Pandemie, vor dem Hintergrund der nun erkennbaren und diskutierten Des- und Falschinformationen und eines zunehmenden Medienpopulismus in den sozialen Medien analytisch deutlich mehr auf empirische fundierte Forschung zur Gesundheitskommunikation und -politik zu setzen [70].
